# Thoracoscopic Resection of a Foregut Duplication Cyst with the Use of a 5-mm Stapling Device in an Infant—A Case Report

**DOI:** 10.1055/s-0042-1742713

**Published:** 2022-03-10

**Authors:** Marc Da Col, Nicolas Regamey, Philipp O. Szavay

**Affiliations:** 1 Department of Pediatric Surgery, Children's Hospital Lucerne, Lucerne, Switzerland; 2 Department of Pediatrics, Pediatric Pulmonology, Children's Hospital Lucerne, Luzern, Switzerland

**Keywords:** foregut duplication cyst, thoracoscopy, stapling device, VATS, pediatric

## Abstract

Esophageal foregut duplication cysts are a rare congenital anomaly predominantly diagnosed in children. With possible growth foregut duplication cysts may cause compression on thoracic or mediastinal structures, respectively. Due to the presence of ectopic gastric mucosa and its potential malignant alteration resection of foregut duplication cysts is recommended. More recently, the use of a thoracoscopic approach for resection has shown to be an advantageous alternative to a conventional open approach. A case of a complete thoracoscopic resection of an esophageal foregut duplication cyst using a 5-mm stapling device is presented.

## Introduction

Esophageal duplication cysts are a rare congenital anomaly predominantly found in children with a male prevalence. Aside from possible compression on the trachea and its expansion into the lung, there is a possibility of malignant alteration of the mucous cells. Therefore, a surgical excision is highly recommended.

Video-assisted thoracoscopic surgery has been established for resection of mediastinal masses in pediatric surgery. 1 2 3 However, there is little evidence in literature on optimal surgical approach for resection of esophageal duplication cysts. This case report aims to present a new approach using a linear stapler for complete resection of an esophageal duplication cyst in pediatric patients.

## Case Report

**Fig. 1:**
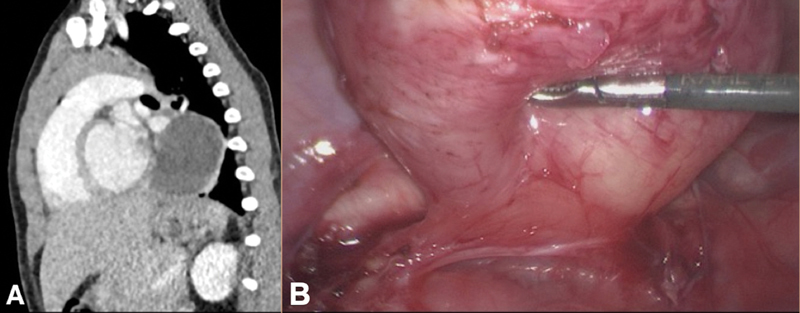
( **A** ) Computed tomography of the chest showing a foregut cyst in the posterior mediastinum in the left hemithorax. ( **B** ) Exposed base of the foregut cyst with crossing vagus nerve at the base of the mass and the descending aorta behind the base of the mass.

A 19-month-old male toddler with prenatal suspected cystic pulmonary mass was referred to our department. At the age of 1 year a contrast magnetic resonance imaging showed a 2 × 2 × 1.5 cm hyperintense fluid-filled cyst on the left paravertebral side with contact to the esophagus in the posterior mediastinum, adherent to the bronchial hilum cranially and to the descendent aorta. A thoracoscopic resection was anticipated. The preoperative computerized tomography showed a progression of the mass to 3.0 × 3.3 × 4.0 cm ( Fig. 1 ).

**Fig. 2:**
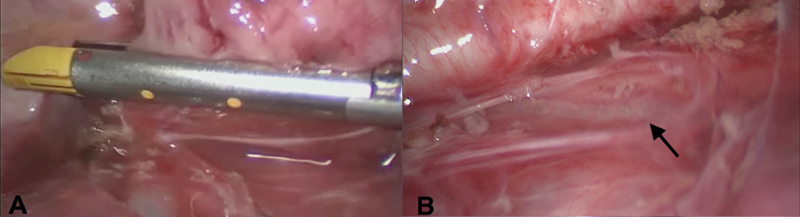
( **A** ) Placement of the stapling device. ( **B** ) Staple line on the esophagus (arrow) after resection.

The patient was operated in a slight prone position with elevated left hemithorax and single-lung ventilation. A 5-mm camera trocar was brought in below the inferior angle of the scapula. Insufflation with 5 mm Hg was initiated. A second and a third 5-mm port were placed ventrocaudal and dorsal. The cyst could be easily identified, then was dissected from the left lower pulmonary lobe with hook electrocautery. Careful dissection of the surrounding pleura along the cystic mass was performed in the same fashion. To the medial side the mass was adherent to the thoracic aorta and the pulmonary hilum. The cyst was dissected in this part again using hook cautery and in addition with a 3-mm vessel sealing device (JustRight, Bolder Surgical, Boulder, Colorado, United States). A broad base connection to the esophagus was displayed ( Fig. 1 ). Macroscopically the mass imposed as a foregut duplication cyst. A complete resection at the base of the duplication cyst could be achieved with the use of a 5-mm stapling device (JustRight, Bolder Surgical) ( Fig. 2 ). There was no indication for an injury to the esophagus. The cyst was removed through the ventrocaudal port site after increasing the port site to 2 cm. A chest drain was not considered to be necessary. Postoperatively there was no sign of a pneumothorax. A nasogastric tube was placed for 24 hours postoperatively. Enteral feeding was started on day 1 postoperatively and the patient was discharged to home on day 4 postoperatively.

Histopathology confirmed a foregut duplication cyst with a two-muscle layer cover and mucinous cells.

## Discussion

Resection through thoracotomy has been the standard of care for excision of foregut duplication cysts. In recent years a thoracoscopic approach has been described for this indication and proven to be safe. 1 4 5 The thoracoscopic approach offers advantages such as reduced postoperative pain, shortening the length of hospital stay, and improved cosmesis. 6 In addition, the morbidity of a thoracotomy can be avoided.

In literature tissue dissecting devices using ultrasound or thermal energy for removal through enucleation or partial resection have been described. 1 6 7 8 9 Kang et al ^9^ described the successful resection of an esophageal duplication cyst using a stapling device in a 53-year-old patient.

As foregut duplication cysts are arising from the esophageal wall they have to be removed entirely without perforating the esophagus. The use of a 5-mm linear stapler to remove a foregut duplication cyst may benefit the patient by minimizing the risk of iatrogenic esophageal rupture and incomplete removal in a small operative field due to its smaller size and therefor can be placed more accurate.

During dissection with meticulous hemostasis and exact placing of the stapling device in the exact plane a clear visualization of the esophagus is of outmost importance for safe and successful resection. 1

Besides single-lung ventilation we preferred positioning the patient in a slightly prone position allowing for a better visualization of the posterior mediastinum. A chest tube should not be placed as standard procedure.

## Conclusion

Thoracoscopic resection of foregut duplication cyst with a 5-mm stapling device proved to be safe and successful in an 18 months old patient.

## Notes

New Insights and the Importance for the Pediatric Surgeon The use of a linear stapler to remove a foregut duplication cyst may benefit the patient by minimizing the risk of iatrogenic esophageal rupture.
